# Epidemiology and survival of non-malignant and malignant meningiomas in middle-aged females, 2004-2018

**DOI:** 10.3389/fonc.2023.1157182

**Published:** 2023-04-26

**Authors:** Junguo Cao, Weijia Yan, Xinyu Hong, Hong Yan

**Affiliations:** ^1^ Shaanxi Eye Hospital (Xi’an People’s Hospital), Affiliated Xi’an Fourth Hospital, Northwestern Polytechnical University, Affiliated Guangren Hospital, School of Medicine, Xi’an Jiaotong University, Xi’an, Shaanxi, China; ^2^ Division of Experimental Neurosurgery, Department of Neurosurgery, University of Heidelberg, Heidelberg, Germany; ^3^ Department of Ophthalmology, University of Heidelberg, Heidelberg, Germany; ^4^ Department of Neurosurgery, The First Hospital of Jilin University, Changchun, China

**Keywords:** SEER Program, meningiomas, incidence, survival, middle-aged females

## Abstract

**Background:**

The incidence of meningioma is disparate to sex: meningiomas are more common in women than in men, especially in middle-aged women. Understanding the epidemiology and survival of middle-aged women with meningiomas would help estimate their public health impacts and optimize risk stratification.

**Methods:**

Data on middle-aged (35–54 years) female patients with meningiomas between 2004 and 2018 were obtained from the SEER database. Age-adjusted incidence rates per 100 000 population-years were calculated. Kaplan-Meier and multivariate Cox proportional hazard models were utilized in the overall survival (OS) analysis.

**Results:**

Data from 18302 female patients with meningioma were analyzed. The distribution of patients increased with age. Most patients were White and non-Hispanic, according to race and ethnicity, respectively. Over the past 15 years, non-malignant meningiomas have shown an increasing trend; however, malignant meningiomas have shown an opposite trend. Older age, Black population, and large non-malignant meningiomas tend to have worse prognoses. Surgical resection improves OS, and the extent of resection is a critical prognostic factor.

**Conclusions:**

This study observed an increase in non-malignant meningiomas and a decrease in the incidence of malignant meningiomas in middle-aged females. The prognosis deteriorated with age, in Black people, and with large tumor size. Additionally, the extent of tumor excision was found to be a significant prognostic factor.

## 1 Introduction

With an incidence rate (IR) of 8.81 per 100 000 person-years, meningioma was the most prevalent primary intracranial tumor, accounting for 38.3% of all brain tumor types recorded in the US from 2013 to 2017 (1). Meningiomas are more likely to affect women than males (1, 2). They are more common in the elderly, especially those aged > 65 years (3). In addition, 90% of meningiomas are supratentorial, and the remaining 10% are spinal and infratentorial (1, 2). Several risk factors for meningioma have been reported, including ionizing radiation and hormonal abnormalities (4, 5). Interestingly, meningioma risk was observed to rise 1.5–1.7 times following breast cancer (6). Meningioma and breast cancer share several key characteristics that might explain their link, including the potential for growth during the early postpartum period, and the expression of progesterone and estrogen receptors (ER) on the cancer cell membranes (7–9). Currently, several molecular and clinical data point to a beneficial correlation between meningioma diagnosis and hormone replacement treatment (10). However, anti-hormonal therapeutic measures have not been proven successful (11). Therefore, exploring the involvement of endocrine hormones in the development of meningioma requires further study (5).

Meningiomas are more common in women than in men, particularly in middle-aged women (2). According to the CBTRUS data, patients aged 35–54 years had the highest female-to-male incidence ratio of 3.29 times (1). In addition, our previous investigation showed that the IR ratio of female-to-male increased with age, peaking at 3.6 at 45-49 years, and then decreased, with an average ratio of 2.1 (2). In particular, the 35-54 age group had a female-to-male ratio exceeds three (2). Therefore, both studies indicate that women between the ages of 35 and 54 years have a higher chance of developing meningioma than men, which merits further study. However, the descriptive incidence and prognosis of meningiomas in middle-aged females have rarely been studied.

The reliable source of cancer epidemiology and survival in the US, Surveillance, Epidemiology and End Results (SEER) Program dataset, covers approximately 48.0% of the population from the US by population-based cancer registries (12). In the current study, the epidemiology and survival of non-malignant and malignant meningiomas in middle-aged females were thoroughly investigated and updated using the SEER dataset in the US from 2004 to 2018.

## 2 Materials and methods

### 2.1 Data collection

The software SEERStat was used to screen meningioma cases based on the SEER database, “Incidence-SEER Research Data, 18 Registries, Nov 2020 Sub (2000–2018)”, which contains data from 18 cancer registries across the US. Several screening criteria were applied in this study. The range of the year was “2004-2018”. Only “woman” sex was selected. Then, the ICD-O-3 codes were applied for the diagnosis of meningioma as follows: non-meningiomas were determined according to the following nine ICD-O-3 codes: 9530/0 (Meningioma, NOS), 9530/1 (Meningiomatosis, NOS), 9531/0 (Meningothelial meningioma), 9532/0 (Fibrous meningioma), 9533/0 (Psammomatous meningioma), 9534/0 (Angiomatous meningioma), 9537/0 (Transitional meningioma), 9538/1 (Clear cell meningioma), and 9539/1 (Atypical meningioma); malignant meningiomas were determined by the following three ICD-O-3 codes: 9530/3 (Meningioma, malignant), 9538/3 (Papillary meningioma), and 9539/3 (Meningeal sarcomatosis). Participants, subjects with unknown or unspecified items were excluded. Finally, 18302 patients fulfilled the selection criteria were included in further research.

### 2.2 Variables for incidence trends analysis

Aged-adjusted incidence rates (IRs) and 95% confidence intervals (CIs) were calculated for non-malignant and malignant meningiomas from 2004 to 2018. The data of middle-aged men were only included in the study of incidence trends by sex to compare with women and highlight the significant disparities; they were not included in the analysis of IRs by race, ethnicity, tumor location, or survival analysis. Based on age, we divided the patients into 4 groups every 5 years (35-39 years, 40-44 years, 45-49 years, and 50-54 years). White, Black, Asian/Pacific Islander (API), and American Indian/Alaska Native (AIAN) were the four racial subgroups. The ethnic groups consisted of Hispanic and non-Hispanic patients. Supratentorial (ICD-O-3 codes 700, 702-714), infratentorial (716-717), and spinal (701, 720-721, 725) tumors were investigated. IR comparisons excluded unknown, undefined, and other categories. Age-adjusted IRs were reported per 100 000 population and normalized to the US population in 2000. To identify trends over time, the annual percentage change (APC) was calculated using Joinpoint Regression Program 4.6.0.0. Only significant changes in APC with P < 0.05 are shown in the graph.

### 2.3 Survival analysis

For middle-aged female patients with non-malignant and malignant meningiomas between 2004 and 2018, survival analyses were performed according to age, race, ethnicity, tumor location and size, and extent of resection. Tumor size was defined as < 3 cm or ≥ 3 cm. According to the SEER site-specific coding rules, the extent of resection was divided into three subgroups: gross total resection (GTR), subtotal resection (STR), and no surgery. AIAN patients with meningiomas and tumor site were excluded from the malignant meningioma survival analysis because of the small sample size. The other categories remained the same as in the IRs analysis, including age, race, ethnicity, and tumor site. Overall survival (OS) in various groups was calculated using Kaplan–Meier model, and the log-rank test was used to investigate differences between subgroups. To identify independent prognostic variables associated with OS, multivariate Cox proportional hazard models were used to calculate hazard ratios (HRs) and 95% confidence intervals (CIs). Statistical significance was set at P < 0.05. Survival analysis was performed using IBM SPSS Statistics version 25 (IBM Corporation, Armonk, New York, USA).

## 3 Results

### 3.1 Baseline patient characteristics

Data from 18302 patients were analyzed. An overview of the initial characteristics and therapeutic processes of these patients is presented in **Table 1**. We observed that 99.29% and 0.71% of patients had non-malignant and malignant meningiomas, respectively. The distribution of patients increased with age, with 12.38% (2266) of patients were between 35-39 years, 20.34% (3722) aged 40-44 years, 29.52% (5402) aged 45-49 years old, and 37.77% (6912) aged 50-54 years old. Most patients were White (13951, 76.23%), followed by Black (2612, 14.27%), and API (1597, 8.73%). Regarding ethnicity, 83.9% of patients (15355) were non-Hispanic. In terms of the location of tumors, most of them were supratentorial (17581, 96.06%), some were spinal (709, 3.87%), and infratentorial meningiomas were rare (12, < 0.1%). Most non-malignant tumors were less than 3 cm (12244, 67.38%), but most of the malignant tumors (87, 66.92%) were larger than 3 cm. For the treatment modality, most patients with non-malignant tumors were not performed operations (9900, 54.48%), but for malignant tumors, most of the patients performed operations (101, 77.69%).

**Table 1:** Patient baseline characteristics.

		Non-malignant		Malignant		All	
		Number	%	Number	%	Number	%
In total	18302	18172	99.29	130	0.71	18302	100
Age	35-39 yrs	2243	12.34	23	17.69	2266	12.38
	40-44 yrs	3693	20.32	29	22.31	3722	20.34
	45-49 yrs	5370	29.55	32	24.62	5402	29.52
	50-54 yrs	6866	37.78	46	35.38	6912	37.77
Race	White	13861	76.28	90	69.23	13951	76.23
	Black	2588	14.24	24	18.46	2612	14.27
	AIAN	140	0.77	2	1.54	142	0.78
	API	1583	8.71	14	10.77	1597	8.73
Ethnicity	Hispanic	2923	16.09	24	18.46	2947	16.10
	Non-Hispanic	15249	83.91	106	81.54	15355	83.90
Site	Supratentorial	17456	96.06	125	96.15	17581	96.06
	Infratentorial	12	0.07	0	0.00	12	0.07
	Spinal	704	3.87	5	3.85	709	3.87
Size	< 3 cm	12244	67.38	43	33.08	12287	67.13
	≥ 3 cm	5928	32.62	87	66.92	6012	32.87
Surgery	No surgery	9900	54.48	29	22.31	9929	54.25
	STR	2573	14.16	33	25.38	2606	14.24
	GTR	5699	31.36	68	52.31	5767	31.51

AIAN, American Indian/Alaska Native; API, Asian/Pacific Islander; GTR, gross total resection; STR, subtotal resection; yrs, years old.

### 3.2 Incidence trends of middle-aged patients with non-malignant and malignant meningioma from 2004 to 2018

#### 3.2.1 Meningioma incidence by age and sex

**Figure 1:**
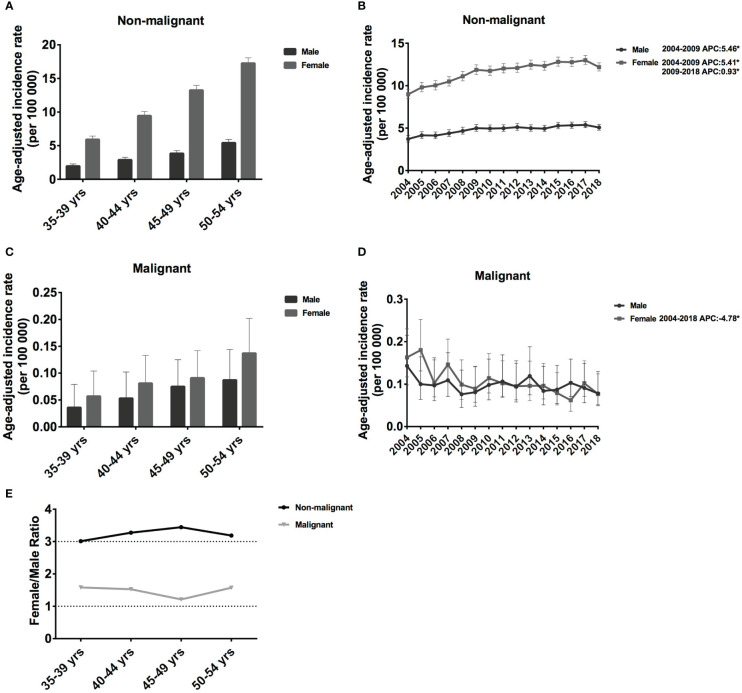
Age-adjusted incidence rates (IRs) and annual percent changes (APCs) by age and sex. IRs by sex and by 5-year age intervals **(A, C)**, APCs by sex over time from 2004–2018 **(B, D)**. **(A, B)**, non-malignant meningioma; **(C, D)**, malignant meningioma; **(E)**, the female to male ratio curve of the IRs by age. *Only show APCs that are significantly different at the P < 0.05 level.

In this study, we investigated the incidence trends in middle-aged female patients with meningioma. However, to better understand the vast differences between females and males, we also discuss the incidence by age and sex. For non-malignant meningiomas in each 5-year age group, the IR grew considerably. IR increased significantly for each 5-year age group, from 1.96 cases per 100 000 population aged 35–39 years (95% CI: 1.70-2.29) to 5.42 cases per 100 000 (95% CI: 4.98-5.92) in age 50-54 years for males, and from 5.92 cases per 100 000 population age 35–39 years (95% CI: 5.46-6.44) to 17.26 cases per 100 000 (95% CI: 16.51-18.07) in age 50-54 years for female (**Figure 1A**). The IR of females was much higher than that of n males, and the IR ratio of females to male was around 3.01-3.44 in non-malignant meningioma (**Figure 1E**). Regarding malignant meningiomas, the IR was much lower in both females and males. In detail, IR increased from 0.036 cases per 100 000 population aged 35–39 years (95% CI: 0.016-0.079) to 0.087 cases per 100 000 (95% CI: 0.053-0.144) in age 50-54 years for male, from 0.057 cases per 100 000 population aged 35–39 years (95% CI: 0.031-0.104) to 0.137 cases per 100 000 (95% CI: 0.094-0.202) in age 50-54 years for female (**Figure 1C**). Interestingly, in contrast to non-malignant meningiomas, the gap by sex was much lower than that of malignant meningioma, and the IR ratio of females to males was around 1.21-1.58 (**Figure 1E**). Therefore, male meningiomas have a substantially lower IR than female meningiomas, especially non-malignant meningioma, and the IR increase with age.

For both males and females, the incidence of non-malignant meningioma increased significantly between 2004 and 2009 (female APC: 5.46% [95% CI: 3.9-7.2], P < 0.001; male APC: 5.41% [95% CI: 3.6-7.6], P < 0.001; **Figure 1B**). However, the growth rate slowed (female APC: 0.93% [95% CI: 0.30-1.60], P = 0.003; **Figure 1B**) from 2009 to 2018. In malignant meningiomas, there was a considerable drop in female prevalence (APC: −4.78% [95% CI: −7.1, −2.3], P = 0.001) from 2004 to 2018 (**Figure 1D**). Therefore, in the past 15 years, the IRs of non-malignant meningiomas have shown an increasing trend, whereas those of malignant meningiomas have shown a decreasing trend.

#### 3.2.2 Middle-aged female meningioma incidence by age and race

**Figure 2:**
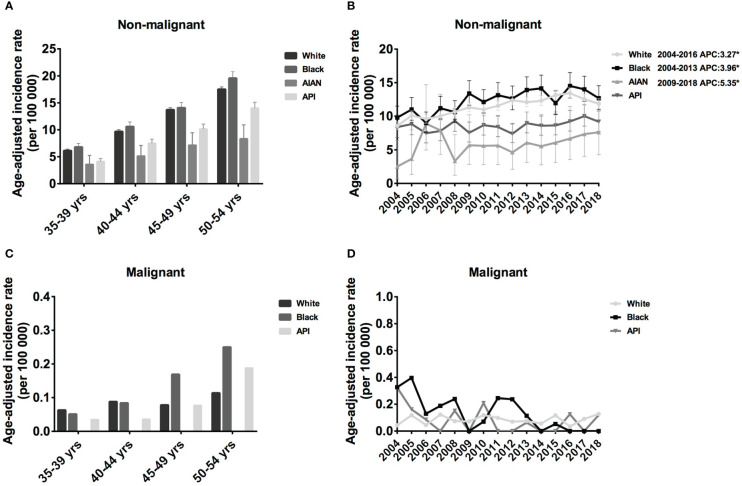
Age-adjusted incidence rates (IRs) and annual percent changes (APCs) by age and race. IRs by race and by 5-year age intervals **(A, C)**, APCs by race over time from 2004–2018 **(B, D)**. **(A, B)**, non-malignant meningioma; **(C, D)**, malignant meningioma. *Only show APCs that are significantly different at the P < 0.05 level.

Here, we only investigated the incidence of meningioma in middle-aged female meningioma. In the Black population, the IR of non-malignant meningioma was slightly higher than that in White patients, followed by API and AIAN. In addition, the IR of all races increased with age, from 6.79 cases per 100 000 population aged 35–39 years (95% CI: 6.14-7.49) to 19.57 cases per 100 000 (95% CI: 18.42-20.76) in age 50-54 years in the Black population, from 6.13 cases per 100 000 population age 35–39 years (95% CI: 5.86-6.41) to 17.50 cases per 100 000 (95% CI: 17.06-17.95) in age 50-54 years in White patients (**Figure 2A**). Regarding malignant meningioma, IR increased with age in Black and API populations but remained relatively stable in white populations. In detail, the IR increased from 0.051 cases per 100 000 population aged 35–39 years (95% CI: 0.010-0.148) to 0.25 cases per 100 000 (95% CI: 0.137-0.490) in the 50-54 years in the Black population, from 0.035 cases per 100 000 population age 35–39 years (95% CI: 0.004-0.126) to 0.188 cases per 100 000 (95% CI: 0.086-0.357) in the 50-54 years in the API (**Figure 2C**). Taken together, our investigation showed that the IR of meningioma was high in the black population and low in the AIAN population for every age group.

Regarding the incidence trends, the IR of non-malignant meningioma showed an increasing trend for all four races from 2004 to 2018; however, malignant meningioma showed the opposite trend. In detail, from 2004 to 2016, the prevalence of White patients with non-malignant meningioma considerably increased (APC: 3.27% [95% CI: 2.5,4.0], P < 0.001), and the IR of Black patients increased until 2013 (APC: 3.96% [95% CI: 1.3,6.7], P = 0.008). From 2009 to 2018, the IR of AIAN patients shown an increasing trend (APC: 5.35% [95% CI: 2.3,8.5], P = 0.004) (**Figure 2B**). Although there was a trend toward a decline in the IR of malignant meningiomas, this shift was not statistically significant (**Figure 2D**).

#### 3.2.3 Middle-aged female meningioma incidence by age and ethnicity

**Figure 3:**
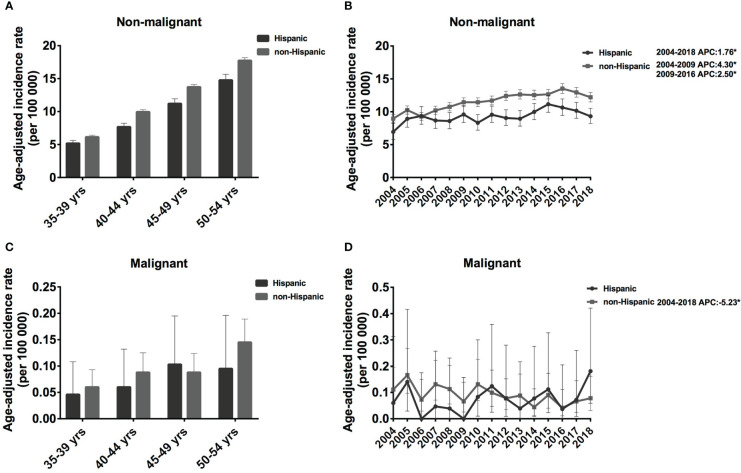
Age-adjusted incidence rates (IRs) and annual percent changes (APCs) by age and ethnicity. IRs by ethnicity and by 5-year age intervals **(A, C)**, APCs by ethnicity over time from 2004–2018 **(B, D)**. **(A, B)**, non-malignant meningioma; **(C, D)**, malignant meningioma. *Only show APCs that are significantly different at the P < 0.05 level.

The IR of the non-Hispanic population was slightly higher than that of Hispanics, and the IR of both populations increased with age. For non-malignant meningioma in Hispanic populations, the IR was 5.18 cases per 100 000 population (5.18 [95% CI: 4.76, 5.63]) in the 35-39 age group and increased continually to 14.76 cases per 100 000 population (14.76 [95% CI: 13.90, 15.67]) in the 50-54 age group. A similar trend was observed in non-Hispanic populations; IR climbed from 6.15 cases per 100 000 population (6.15 [95% CI: 5.89, 6.42]) to 17.74 cases per 100 000 population (17.74 [95% CI: 17.32, 18.17]) (**Figure 3A**). However, the IR of malignant meningioma was much lower than that of non-malignant meningiomas, which was approximately 0.046 [95% CI: 0.015, 0.108] to 0.103 [95% CI: 0.047, 0.195] cases per 100 000 population in Hispanic, and 0.06 [95% CI: 0.037, 0.093] to 0.145 [95% CI: 0.109, 0.189] cases per 100 000 population in non-Hispanic patients (**Figure 3C**).

From 2004 to 2018, non-malignant meningiomas showed an increasing trend in both the Hispanic and non-Hispanic populations. Specifically, the IR of Hispanic patients increased significantly from 2004 to 2018 (APC: 1.76% [95% CI: 0.7-2.9], P = 0.004). In addition, the IR of non-Hispanic patients increased significantly from 2004-2009 (APC: 4.30% [95% CI: 1.1-7.6], P = 0.015), and the rate of increase slowed from to 2009-2016 (APC: 2.50% [95% CI: 0.1-4.9], P = 0.041) (**Figure 3B**). In contrast, for malignant meningioma in non-Hispanic populations, the IR showed a decreasing trend from 2004 to 2018 (APC: -5.23% [95% CI: -9.0, -1.3], P = 0.014) (**Figure 3D**). Therefore, the IRs of non-meningiomas have shown an increasing trend in both non-SHL and SHL populations, while malignant meningiomas have shown a decreasing trend in non-SHL populations in the past 15 years.

#### 3.2.4 Middle-aged female meningioma incidence by age and tumor location

**Figure 4:**
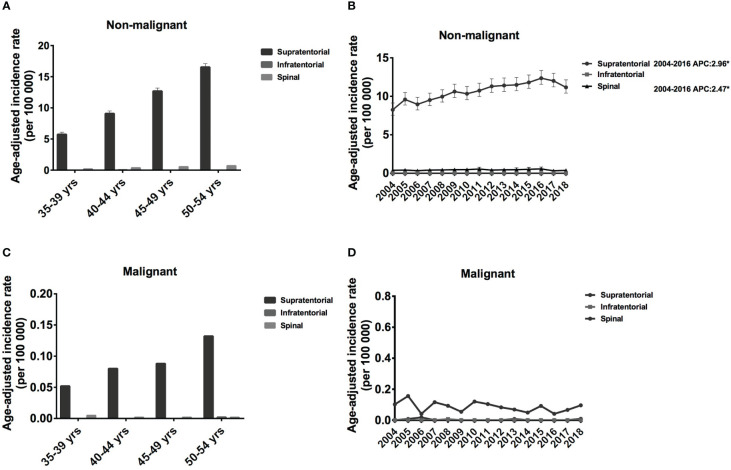
Age-adjusted incidence rates (IRs) and annual percent changes (APCs) by age and tumor location. IRs by tumor location and by 5-year age intervals **(A, C)**, APCs by tumor location over time from 2004–2018 **(B, D)**. **(A, B)**, non-malignant meningioma; **(C, D)**, malignant meningioma. *Only show APCs that are significantly different at the P < 0.05 level.

Regarding the tumor location, most of them were in supratentorial (17581, 96.06%), some located in spinal (709, 3.87%), and infratentorial meningiomas were rare (0.1%). Every five years, the IRs for supratentorial non-malignant meningiomas increased significantly, from 5.73 [95% CI: 5.43-6.09] to 16.54 [95% CI: 16.03-17.10] cases per 100 00 population (**Figure 4A**). A similar trend was observed for supratentorial malignant meningioma; however, IR was much lower, ranging from 0.052 [95% CI: 0.028-0.13] to 0.132 [95% CI: 0.089-0.23] cases per 100 00 population (**Figure 4C**). Regarding spinal meningioma, the IR was around 0.183 [95% CI: 0.146-0.252] to 0.713 [95% CI: 0.631- 0.826] for non-malignant meningioma and 0.002 [95% CI: 0.001-0.36] to 0.05 [95% CI: 0.001-0.04] for malignant meningioma (**Figures 4A, C**). Therefore, the IR of supratentorial meningiomas is significantly higher than that of spinal and infratentorial meningiomas.

From 2004 to 2016, the IR increased significantly for both supratentorial (APC: 2.96% [95% CI: 2.3-3.6], P < 0.001) and spinal non-malignant meningioma (APC: -2.47% [95% CI: 0.5-4.5], P = 0.02) (**Figure 4B**). Although supratentorial and spinal malignant meningiomas showed a decreasing trend from 2004-2018, this change was not statistically significant (**Figure 4D**). Therefore, the IRs of supratentorial non-malignant meningiomas have shown an increasing trend, while malignant meningiomas have shown a decreasing trend over the past 15 years.

### 3.3 Kaplan–Meier results

**Figure 5:**
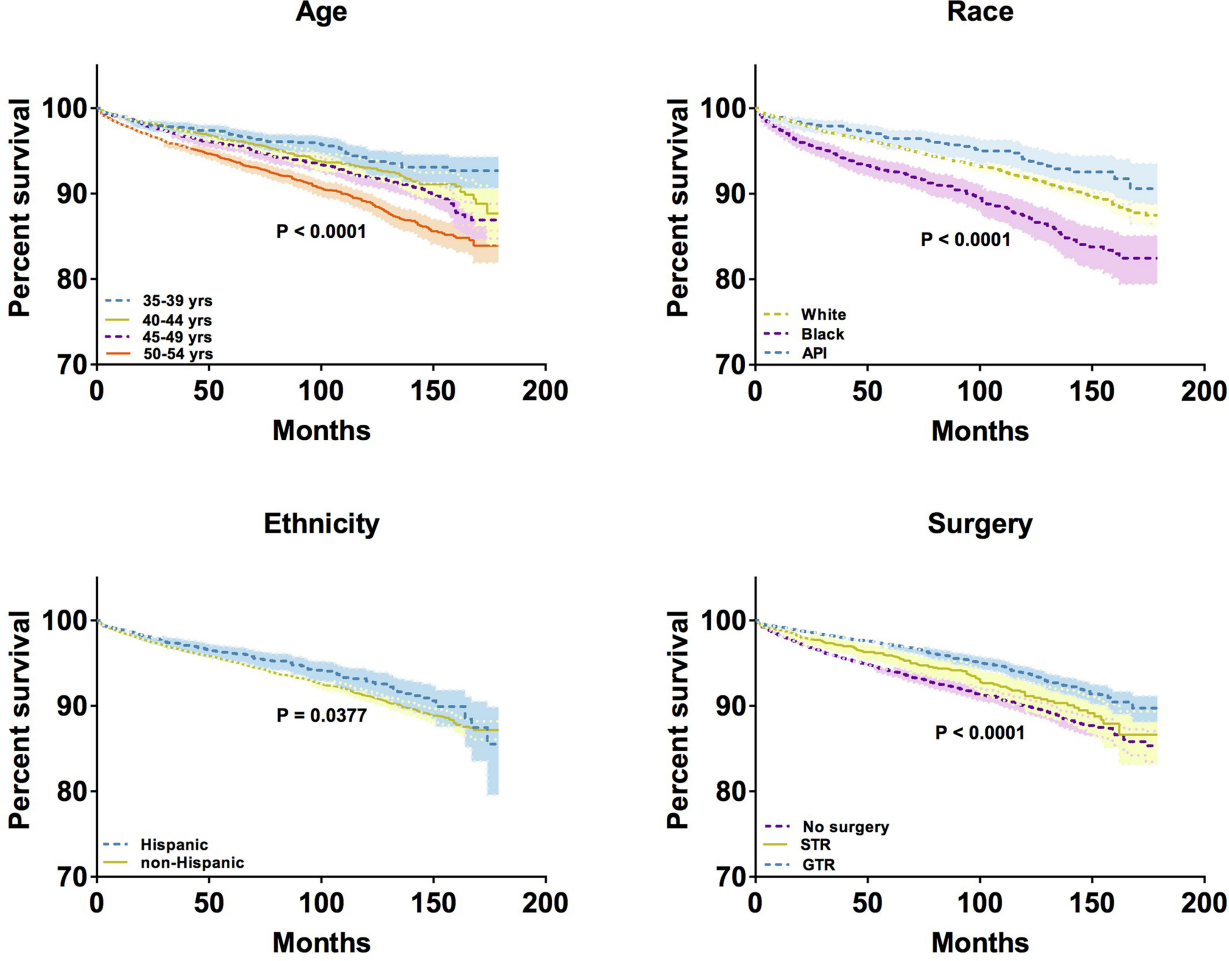
Kaplan–Meier analysis by age, race, ethnicity, and surgical treatment. * Only show significant differences in overall survival at the P < 0.05 level.

To compare the overall survival of middle-aged female patients with meningioma with respect to different variables, the Kaplan–Meier model was used to perform survival analysis. We observed significant differences in overall survival by age (P < 0.0001), race (P < 0.0001), ethnicity (P < 0.0001), and surgical resection status (P < 0.0001), but not by tumor location and tumor size (**Figure 5**). The 10-year survival rates of patients aged 35-39, 40-44, 45-49, and 50-54 years were 93.92%, 93.04%, 91.96%, and 89.02%, respectively. In terms of 10-year survival rates of patients differing in different races, the highest was in API patients (94.06%), followed by white patients (91.91%), and black patients (87.30%). In addition, the 10-year survival rates of patients differed by ethnicity: 92.90% in Hispanic patients and 91.17% in non-Hispanic patients. Regarding the extent of resection, the 10-year survival rates of patients who underwent GTR and STR were 93.83% and 91.14%, respectively; however, the rate was 89.98% for patients who did not undergo any surgery. Taken together, older age, Black race, non-Hispanic ethnicity, and high extent of resection in middle-aged female patients with meningioma tend to have longer overall survival.

### 3.4 Results from multivariable cox proportional hazards model

Next, we investigated the association between demographic variables and overall survival in non-malignant meningiomas using multivariate Cox proportional hazards regression models (**Table 2**). After controlling for factors, the model showed that all the following factors, including age, race, tumor size, and extent of resection, had a significant impact on survival in non-malignant meningiomas. Except for patients of 40-44-year-olds, the risk of mortality increased significantly with each increase in the 20-year age group when compared to a group of 35-39 years old: patients of 45-49 years old had a 1.51 times greater risk of mortality (HR: 1.51 [95% CI:1.20-1.90, P < 0.001], 50-54 years old patients were 2.09 times (HR: 2.09 [95%CI: 1.67-2.60], P < 0.001). Black patients had a 71.5% higher risk of mortality (HR: -1.715 [95% CI: 1.490-1.974], P < 0.001) than white patients did. In terms of tumor size, patients with a larger tumor ≥ 3 cm had a 45.1% increased risk of mortality (HR: 1.45 [95% CI: 1.24-1.70], P < 0.001) than patients with a tumor <3 cm. Regarding the extent of resection, STR and GTR operations reduced death risk by 23.7% and 44.4%, respectively (STR: HR: 0.76 [95% CI: 0.63-0.92], P = 0.004; GTR: HR: 0.56 [95% CI: 0.48-0.65], P < 0.001). In contrast to the multivariable Cox proportional hazards for non-malignant meningioma, there were no statistically significant differences in survival in malignant meningioma according to any of the analyzed variables.

**Table 2:** Hazard rations (HR) and 95% confidence intervals (CIs) for patient demographics and treatment modalities in Multivariable Cox Proportional Hazards Model for middle-aged female non-malignant meningiomas survival.

Non-malignant			Kaplan-Meier Results			Multivariable Cox Proportional Hazards Model		
			P-value			HR	95%CI	P-value
Age	35-39 yrs		< 0.001			Reference		
	40-44 yrs					1.264	0.988-1.619	0.063
	45-49 yrs					1.507	1.197-1.897	< 0.001
	50-54 yrs					2.085	1.674-2.596	< 0.001
Race	White		< 0.001			Reference		
	Black					1.715	1.490-1.974	< 0.001
	AIAN					1.133	0.587-2.186	0.710
	API					0.815	0.644-1.031	0.088
Ethnicity	Hispanic		0.123			Reference		
	Non-Hispanic					1.009	0.850-1.197	0.922
Location	Supratentorial		0.160			Reference		
	Spinal					0.906	0.647-1.268	0.563
Size	< 3cm		0.467			Reference		
	≥ 3cm					1.451	1.239-1.698	< 0.001
Surgery	No surgery		< 0.001			Reference		
	STR					0.763	0.633-0.919	0.004
	GTR					0.556	0.478-0.647	< 0.001

AIAN, American Indian/Alaska Native; API, Asian/Pacific Islander; GTR, gross total resection; STR, subtotal resection; yrs, years old.

## 4 Discussion

Meningiomas are the most common intracranial tumor (1). Although most meningiomas are benign, approximately 20% of patients have aggressive meningiomas with poor a prognosis (1). Many studies have confirmed that the incidence of meningioma is disparate by sex, and the female-to-male ratio of IR is over than three in the 35–54-year group (1, 2, 13, 14). However, the underlying mechanism remains unclear. To date, only a few studies have focused on this population. The current study used a 15-year time period dataset to offer an in-depth update on the epidemiology of both nonmalignant and malignant meningiomas in middle-aged females. Following earlier findings, we examined data from 18 302 individuals and discovered that 99.3% of patients had non-malignant meningiomas and 0.7% had malignant meningiomas. A total of 159,038 meningioma cases between 2013 and 2017 were documented by Quinn et al. who reported that the percentages of non-malignant and malignant meningiomas were 98.9% and 1.1%, respectively (1). The present study only included middle-aged female patients, but not elderly patients, which explains why the percentage of malignant meningiomas was lower.

Recent studies have shown that during the past 20 years, the incidence of CNS malignancies has reduced (1, 15). However, the incidence patterns are far more varied when histology-specific studies are carried out (5, 16, 17). Furthermore, given that the female-to-male incidence rate ratios were the highest in those aged 35-54 years old, it is important to carefully examine meningioma incidence and survival in middle-aged females. Over the past two decades, researchers have been investigating the sex hormone dependence of meningiomas (18). The accelerated growth rate during pregnancy and the luteal phase of the menstrual cycle, as well as the greater occurrence in females, point to the possibility that sex hormones may play a role in the development of meningiomas (19). A number of researchers have reported a correlation between meningioma and researchers (6). Additionally, research has demonstrated that meningiomas express progesterone, estrogen, and androgen receptors (19–24). In contrast, the occurrence of meningiomas in women cannot be explained by variations in sex hormone receptors, according to Korhonen et al., who reported no difference in sex hormone receptor expression between sex and age groups (25). Progesterone and antiprogesterone have been reported to control meningioma growth *in vitro*; however, the moderate effects of anti-progesterone on patient outcomes point to a more nuanced interaction between sex hormones and meningioma growth (18). Therefore, the mechanism of sex discrimination in meningiomas remains controversial (26, 27). Exploring biomarkers and subsequent therapeutic agent is a promising approach for the treatment of meningiomas. Simultaneously, understanding the epidemiology and prognostic factors of middle-aged meningiomas can help estimate the public health impact and optimize risk stratification and treatment strategies.

Herein, we observed that the IR of meningioma increased with age, and the IR of 50-54 years was 2.92 and 2.77 times of IR in 35-39 years for females and males, respectively. Many research also reported that the age maybe a risk factor of meningioma, since the incidence of meningioma increased with age, especially for the patients over 60 years old (1, 14, 28–30). Mechanistically, evidence of aging-related genome-wide DNA methylation alterations in meningiomas may explained this phenomenon (31–33). Furthermore, most patients were White and non-Hispanic, accounting for 76.3% and 83.9%, respectively. Regarding the location, the majority were supratentorial, some were spinal, and infratentorial meningiomas were rare. Most non-malignant tumors were < 3 cm in size, whereas most malignant tumors were ≥ 3 cm in size. In terms of treatment modality, most patients with non-malignant tumors did not undergo surgery; however, most patients underwent surgery for malignant tumors.

Based on the trend of middle-aged female meningiomas over the past 15 years, we observed an increasing number of cases of non-malignant meningiomas. There are some possible hypotheses may explain this increasing trend, including the aging of the population, the development of diagnostic technologies, and the increase frequency of seeking medical care (1, 30, 34, 35). However, the trend of malignant meningiomas is reversed, with a decreasing incidence. Probably due to the development of diagnostic technology and the increase in the frequency of people seeking medical treatment, many meningiomas are diagnosed and treated at an early stage, avoiding progression to a high level (1, 3). Furthermore, the updated WHO classification guidelines published in 2007 may also contribute to changes in the trend of meningioma (3). Non-malignant meningioma shows an increasing trend, and malignant meningiomas show a decreasing trend among middle-aged females over the past 15 years in the US.

Regarding the survival analysis, we discovered several characteristics related to a worse survival rate in patients with non-malignant meningiomas. In contrast to non-malignant meningiomas, there were no statistically significant differences in survival in malignant meningiomas according to any of the analyzed variables. Kaplan-Meier analysis showed that older patients tended to have worse overall survival. In multivariable Cox proportional hazards regression analysis, the risk of mortality increased significantly with each increase in the 20-year age group when compared to a group of 35-39 years old. From the current data, older age is a major risk factor for poor prognosis, which has also been confirmed in many research (36, 37). Furthermore, the Black population tended to have worse survival rates, and the risk of mortality was 71.5% higher than that of White patients. Black and White populations in the United States were analyzed for mortality trends from 1900 to 2010 by Robert A. et al., who provided a number of explanations for why Black people have a lower survival rate, including social and environmental factors, biological and behavioral factors, preventive and therapeutic interventions, and access to them (38). However, racial variations in tumor activity may be influenced by molecular or epigenetic factors. Further research is required to understand the processes driving these disparities.

According to the results of the multivariable Cox proportional hazards regression model, patients with a tumor ≥ 3 cm had a 45.1% greater chance of death than those with a tumor < 3 cm. This finding was supported by previous research, which showed that tumor size is among the most crucial prognostic markers affecting tumor recurrence and patient survival. Interestingly, female meningiomas have been reported to grow and become clinically symptomatic during pregnancy, requiring the attention of neurosurgeons (7, 9). This may be related to pregnancy-related potentially reversible hemodynamic changes in and around tumor edema and may also be related to hormone-induced cell proliferation, which is still debated and require further investigation (39).

Regarding the extent of resection, we observed that STR and GTR procedures decreased the mortality rate by 23.7% and 44.4%, respectively, compared with no surgery. Further analysis demonstrated that any two of the three subtypes had substantial differences in OS, indicating that the OS of patients in the no surgery group was worse than in the STR group, and that STR was worse than GTR. Taken together, the extent of resection is a critical prognostic factor for middle-aged female patients with meningiomas. Here, we were unable to analyze data on malignant meningiomas. As this topic only analyzes meningiomas in middle-aged women, malignant meningiomas occur frequently in elderly patients, limiting the number of malignant meningiomas (2). Because the prognosis of middle-aged women with non-malignant meningiomas is generally better, the cause of death may be other factors. OS analysis has certain limitations, and competitive survival analysis may help to dissect more accurate patterns (2). However, the collected data lacked sufficient cause-specific survival data for competitive survival analysis.

## 5 Conclusions

The incidence patterns and survival of meningiomas according to all demographics among a particular group middle-aged female, are thoroughly reviewed in this study. Despite several limitations, we identified that older age, Black race, and large tumor size may be significantly worse prognostic variables. Our findings also suggest that tumor excision can significantly increase the survival of middle-aged female patients with meningiomas. In the future, we should not only analyze data based on clinical and demographic characteristics but also explore molecular signs such as epigenetic changes or genetic mutations in meningiomas. Importantly, exploring the molecular mechanism of the high incidence of meningioma in middle-aged women, and based on this discovery of therapeutic drugs for meningioma, will be the future development direction.

## Data availability statement

The original contributions presented in the study are included in the article/supplementary material. Further inquiries can be directed to the corresponding author.

## Ethics statement

SEER data is publicly available and de-identified. Cases were collected from the SEER database and were analyzed anonymously in this study. Therefore, no ethical review or additional informed consent was required.

## Author contributions

Conceptualization, JC, HY, and XH; methodology, JC and WY; software, JC and WY; validation, JC and WY; formal analysis, JC and WY; investigation, JC and WY; resources, HY and XH; data curation, JC; writing-original draft preparation, JC; writing--review and editing, JC; visualization, JC; supervision, HY and XH; project administration, HY and XH; funding acquisition, HY and XH. All authors have read and agreed to the published version of the manuscript. All authors contributed to the article and approved the submitted version
